# A four-dimensional model for the Ba–Ti–O dodeca­gonal quasicrystal

**DOI:** 10.1107/S205252062200227X

**Published:** 2022-03-24

**Authors:** Tsunetomo Yamada

**Affiliations:** a Faculty of Science, Department of Applied Physics, Tokyo University of Science, 6-3-1 Niijuku, Katsushika-ku, Tokyo 125-8585, Japan

**Keywords:** quasicrystal, approximant, high-dimensional crystal, oxide, thin film

## Abstract

A four-dimensional (4D) model is presented of the first oxide dodecagonal quasicrystal found in a Ba–Ti–O ultra-thin film on a Pt(111) single-crystal substrate. The 4D model, with a 4D dodecagonal lattice constant *a*_*d*_ = 8.39 Å, was derived by considering a tile decoration model of dodecagonal Niizeki–Gähler tiling composed of squares, triangles and 30° rhombuses. The model consists of four kinds of occupation domain, and 4D positional vectors defining the shape of each occupation domain are given. Moreover, the atomic arrange­ment of two Ba–Ti–O periodic approximants, the sigma-phase approxi­mant and a 25.6 Å approximant were derived from the 4D model by the introduction of linear phason strains.

## Abstract 2

A four-dimensional structure model for the Ba–Ti–O dodecagonal quasicrystal is described.

## 1. Introduction

Quasicrystals (QCs) are long-range-ordered solids that exhibit self-similar diffraction patterns incompatible with translational symmetry (Shechtman *et al.*, 1984 ▸; Levine & Steinhardt, 1984 ▸). The first dodecagonal quasicrystal (DDQC) was found in small particles of an Ni–Cr alloy (Ishimasa *et al.*, 1985 ▸). DDQCs have since been observed not only in alloys but also in various systems, including liquid crystals, cylindrical polymers, colloids and nanoparticles [see, for example, Ishimasa (2011 ▸), Dotera (2011 ▸), and references therein].

The first oxide QC was reported recently by Förster *et al.* (2013 ▸) in a Ba–Ti–O ultra-thin film on a Pt(111) single-crystal substrate. The oxide QC was identified as a DDQC by observation of a 12-fold pattern in low-energy electron diffraction (LEED) images. In addition, the arrangement of protrusions observed in scanning tunneling microscopy (STM) images corresponds to dodecagonal Niizeki–Gähler tiling (NGT), which is composed of three tiles, *i.e.* a triangle, square and 30° rhombus (hereafter rhombus) (Niizeki & Mitani, 1987 ▸; Gähler, 1988 ▸), with an edge length of 6.85 Å. Atomic positions determined based on the STM images were statically analyzed and compared with the NGT by Schenk *et al.* (2019*b* ▸). A second DDQC was more recently observed in an Sr–Ti–O ultra-thin film on a Pt(111) substrate (Schenk *et al.*, 2017 ▸). The oxide DDQCs form on a periodic threefold sub­strate; therefore, the formation and propagation mechanism of the quasiperiodic long-range order are of significant interest. However, knowledge of the atomic structure of the DDQCs is crucial to understand the mechanism.

A tile decoration model of the Ba–Ti–O DDQC was proposed and investigated in detail by Cockayne *et al.* (2016 ▸). This model consists of three decorated tiles in the NGT, which leads to a stoichiometry of Ba_0.37_TiO_1.55_. The stability of the atomic structure was also investigated with hypothetical approximants (APs) to the DDQC using density functional theory (DFT) calculations, where the DFT-relaxed structures re­tain­ed the ideal tile geometries. In addition, a simulated STM image based on the relaxed atomic structure of a hypothetical AP reproduced the experimental image, which indicates that the protrusions observed in the STM images are Ba atoms.

In higher-dimensional descriptions of DDQCs, the quasiperiodic atomic structure can be obtained as a three-dimensional (3D) section of a five-dimensional (5D) periodic structure that consists of so-called *occupation domains* (ODs). The OD has a two-dimensional (2D) shape defined in the 2D complementary space called the perpendicular space 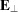 , which is perpendicular to the 3D real space, called the parallel space, 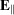 [see, for example, Yamamoto (1996 ▸), Janssen *et al.* (2007 ▸) and Steurer & Deloudi (2009 ▸)]. The 5D structure has two lattice constants, *a*_*d*_ and *c*, and the atomic structure has a period along the *c* axis. The first 5D model was derived for a stable Ta–Te DDQC, which consists of fractal ODs (Yamamoto, 2004 ▸). The Ba–Ti–O DDQC was formed with monolayer thickness (Zollner *et al.*, 2020*a* ▸); therefore, we regard its atomic structure as a 2D structure. In such a case, the atomic structure is obtained as a 2D section of a four-dimensional (4D) structure in a 4D subspace orthogonal to the *c* axis. The higher-dimensional description allows atomic coordinates in the QC structure to be described with finite structural parameters, including 4D coordinates of the ODs and atomic displacement parameters in 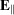 and 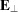 ; therefore, the con­struction of the 4D model is crucial to analyze the atomic structure of DDQCs. Here, we derive a 4D model for the Ba–Ti–O DDQC based on the tile decoration model of Cockayne *et al.* (2016 ▸).

## 2. A simple layer of Niizeki–Gähler tiling

**Figure 1:**
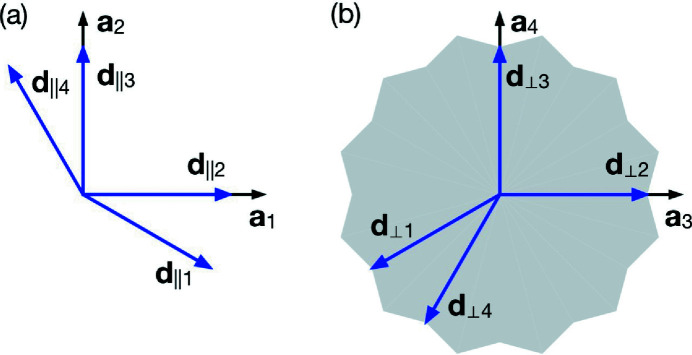
Projection of the unit vectors of the 4D dodecagonal lattice, **d**_*i*_ (*i* = 1,2,3,4), onto (*a*) 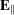 and (*b*) 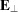 . The grey area represents the OD for NGT, *i.e.* *C*_*a*_ in the literature (Gähler, 1988).

In this section, we briefly describe a simple 4D model which places atoms at face-centre positions of the NGT (Gähler, 1988 ▸). The coordinate system used in this study is based on that described in the literature (Yamamoto, 1996 ▸). The unit vectors of the 4D dodecagonal lattice **d**_*i*_ (*i* = 1,2,3,4) are written using unit vectors in 2D 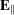 , **a**_1_, **a**_2_, and 2D 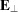 , **a**_4_, **a**_5_, as 

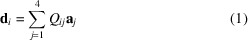

with 

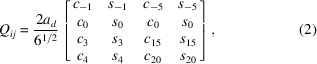

where *a*_*d*_ is the lattice constant of the 4D dodecagonal lattice, and *c*_*n*_ and *s*_*n*_ are 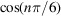 and 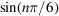 , respectively. The projection of the **d**_*i*_ onto 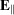 and 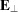 is shown in Fig. 1 ▸. A 4D positional vector **x** = (*x*,*y*,*z*,*u*) is represented by using the **d**_*i*_, and the perpendicular-space component of the **x** is represented by subscript 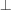 (the parallel-space component is represented by the subscript 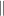 ). The coordinates in 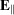 and 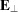 are given by 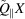 and 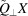 , respectively, where 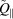 and 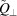 are the upper and lower 2 × 4 part of the transposed matrix of *Q* in equation (2), respectively, and *X* is a transposed matrix of (*x*,*y*,*z*,*u*).

Hereafter, we consider the 4D model with a lattice constant of the 4D dodecagonal lattice *a*_*d*_ equal to 8.39 Å. This lattice constant corresponds to the edge length of the tile elements given by 2*a*_*d*_/6^1/2^, which is equal to 6.85 Å and corresponds to the observed edge length in the STM images of the Ba–Ti–O DQC (Förster *et al.*, 2013 ▸).

The vertex positions of the NGT are obtained from an OD that is denoted as *C*_*a*_ in the literature (Gähler, 1988 ▸). The shape of *C*_*a*_ is shown in Fig. 1 ▸(*b*). *C*_*a*_ is situated at the vertex position of the 4D dodecagonal lattice with site symmetry 12*mm*, provided that a plane group of the resulting tiling of *p*12*mm* is assumed. The asymmetric part of *C*_*a*_ is defined as a triangle where the vertices are represented by 4D positional vectors (0, 0, 0, 0), (−3^1/2^, 3^1/2^, 0, 0) 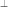 /3 and (0, 1, 0, 0) 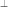 in a unit of 2*a*_*d*_/6^1/2^.

**Figure 2:**
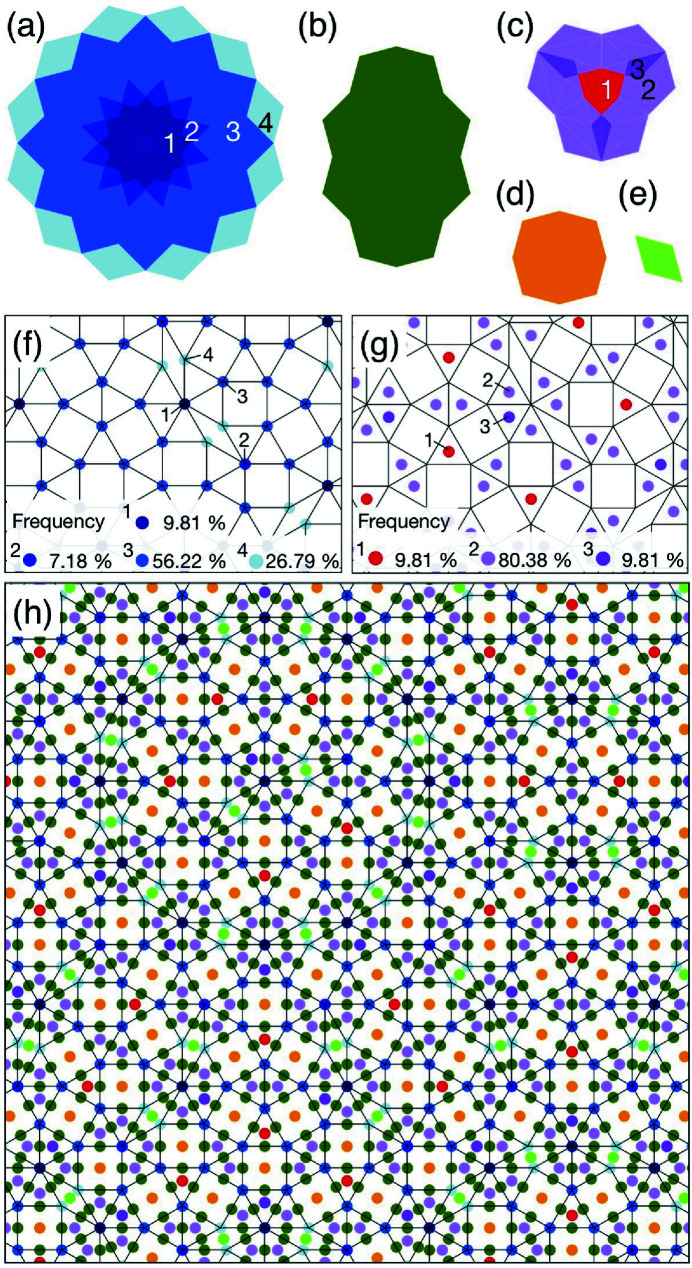
NGT with edge-centre and face-centre atoms. The ODs of the (*a*) vertex, (*b*) edge-centre, (*c*) face-centre of a triangle, (*d*) face-centre of a square and (*e*) face-centre of a rhombus. The divisions assigned by 1–4 in part (*a*) generate vertices in the same local configuration in part (*f*), and the divisions assigned by 1–3 in part (*c*) generate triangles in the same local configuration in part (*g*). The rates in parts (*f*) and (*g*) are the frequencies of each local configuration. The positions derived from the ODs in parts (*a*)–(*d*) are represented by the same colour in part (*h*).

Fig. 2 ▸ shows a simple representation of the NGT at the vertex, edge-centre and face-centre positions. The OD in Fig. 2 ▸(*a*) generates the vertices of the tiling, which corresponds to *C*_*a*_. *C*_*a*_ is subdivided into four parts which are assigned to a1, a2, a3 and a4, so that they distinguish four local configurations present in the tiling, as shown in Fig. 2 ▸(*f*). They distinguish the vertex positions as following; (1) a vertex position derived by OD a1 shares five triangles and two rhombuses; (2) a vertex position by OD a2 shares four triangles, one square and one rhombus; (3) a vertex position by OD a3 shares three triangles and two squares; and (4) a vertex position by OD a4 shares two triangles, one square and one rhombus. The areas of the asymmetric part of ODs a1–4 are [9 − 5(3)^1/2^]/36, [7(3)^1/2^ − 12]/18, [21 − 11(3)^1/2^]/36 and [2(3)^1/2^ − 3]/18; therefore, the frequencies of each local environment are determined as approximately 9.81, 7.18, 56.22 and 26.79%, respectively. Here, the area of each OD is divided by *a_d_* ^2^.

The ODs in Figs. 2 ▸(*b*)–2 ▸(*e*) generate the edge-centre and face-centre of each triangle, the face-centre of each square and the face-centre of each rhombus, respectively. The OD in Fig. 2 ▸(*c*) is subdivided into three parts, which are assigned to c1, c2 and c3 so that they distinguish three local configurations present in the tiling, as shown in Fig. 2 ▸(*g*). They distinguish the triangles as following; (1) a triangle with a face-centre position derived by OD c1 shares no edge with other triangles; (2) a triangle with a face-centre position derived by OD c2 shares one edge with another triangle; and (3) a triangle with a face-centre position derived by OD c3 shares two edges with two other triangles. The areas of the asymmetric part of ODs c1, c2 and c3 are [9 − 5(3)^1/2^]/36, [2(3)^1/2^ − 3]/6 and [9 − 5(3)^1/2^]/36; therefore, the frequencies of each triangle are determined as approximately 9.81, 80.38 and 9.81%, respectively. Here, the area of each OD is divided by *a_d_* ^2^. The positions derived from the ODs in Figs. 2 ▸(*a*)–(*d*) are represented by the same colour in Fig. 2 ▸(*h*), and the Wyckoff positions, site symmetry, coordinates and ODs are summarized in Table 1 ▸, based on the Wyckoff positions of 5D dodecagonal space groups (Yamamoto, 2021 ▸). The 4D positional vectors that define the asymmetric part of ODs a–e are listed in Table 2 ▸. The ODs a–e can be derived from their asymmetric parts by applying the symmetry operations of their respective site-symmetry group.

## 3. 4D model of Ba–Ti–O DDQC

**Figure 3:**
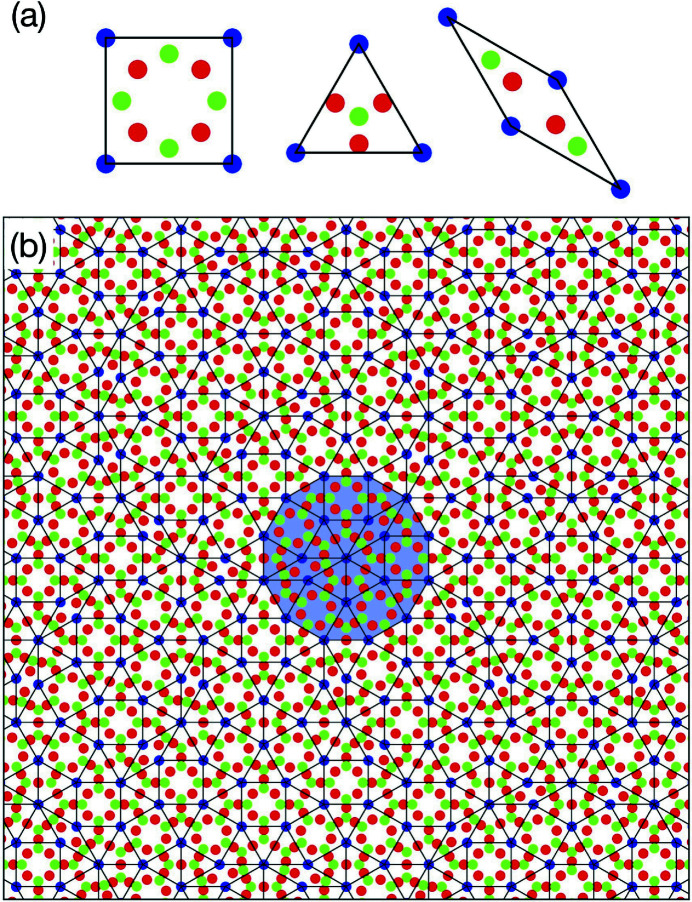
Atomic structure of the Ba–Ti–O DDQC. (*a*) Decoration of square, triangle and rhombus tiles proposed by Cockayne *et al.* (2016). (*b*) Atomic arrangement obtained from the 4D model. Circles drawn in lime, red and blue indicate Ti, O and Ba atoms, respectively. A dodecagon characteristic of the NGT is highlighted by the translucent blue colour.

To derive a 4D model of the Ba–Ti–O DDQC, we take into account the tile model proposed by Cockayne *et al.* (2016 ▸), as shown in Fig. 3 ▸(*a*). The model has the following features. First, the Ba atoms are situated at each vertex position. Second, the Ti atoms are located at the face-centres of each triangle, four positions in each square and two positions in each rhombus. Third, the O atoms are located at four positions in each square and two positions in each rhombus. The O atoms are located at three positions of each triangle; however, the position is dependent on the local environment of the triangle. When two triangles are neighbouring and sharing an edge, the O-atom positions near the edge in each triangle merge into one at a position close to the sharing edge (Cockayne *et al.*, 2016 ▸).

Considering the tile model, the 4D model is constructed using the ODs presented in Fig. 2 ▸. First, the vertex position occupied by Ba is generated from the OD in Fig. 2 ▸(*a*) at (0,0,0,0), and the face centre of each triangle occupied by Ti is generated from the OD in Fig. 2 ▸(*d*) at (0,2,0,1)/3 and its equivalent positions (6*a*). Second, the two positions occupied by Ti and the other two positions by O in each rhombus are generated from the OD in Fig. 2 ▸(*e*). To generate these positions, the OD must be shifted from (1,0,0,1)/2 by ±(*x*_1_,0,0,−*u*_1_) 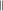 for Ti and by ±(*x*_2_,0,0,−*u*_2_) 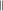 for O along 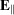 . Third, the four positions occupied by Ti in each square are generated from the OD in Fig. 2 ▸(*d*). This OD must be shifted from (0,1,1,0)/2 by ±(0,*y*_3_,0,0) 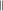 and by ±(0,0,*z*_3_,0) 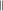 to generate the four positions. Similarly, another four positions occupied by O in each square are obtained by shifting the OD in Fig. 2 ▸(*d*) by ±(0,*y*_4_,*z*_4_,0) 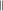 and by ±(0,*y*_4_,−*z*_4_,0) 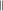 from (0,1,1,0)/2. The positions occupied by O in each triangle are generated from either OD c1, c2 or c3 in Fig. 2 ▸(*c*) shifted from (0,2,0,1)/3. The shifts of OD c1 are −(0,*y*_5_,0,*u*_5_) 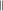 , (0,*y*_6_,0,*u*_6_) 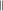 and (0,−*y*_7_,0,*u*_7_) 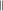 , those of OD c2 are −(0,1,0,2) 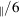 , (0,*y*_6_,0,*u*_6_) 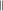 and (0,−*y*_7_,0,*u*_7_) 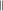 , and those of OD c3 are (0, 2, 0, 1)/3 by −(0, 1, 0, 2) 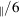 , (0, 2, 0, 1) 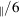 and (0,−*y*_7_, 0, *u*_7_) 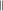 . The coordinates of the ODs in the 4D model are summarized in Table 3 ▸.

The positions obtained by OD c2 and c3 shifted by −(0, 1, 0, 2) 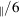 are generated at the edge centre of the triangle at (0, 1, 0, 0)/2 + (0, 1, 0, 2) 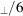 because (0, 1, 0, 2) 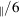 is equivalent to (0, 1, 0, 2)/6 − (0, 1, 0, 2) 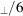 . Similarly, the positions obtained by OD c3 shifted by (0, 2, 0, 1) 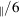 are generated on the edge-centre of the triangle at (0, 2, 0, 1)/2 − (0, 2, 0, 1) 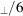 . Therefore, these positions should be half-occupied by O with an occupation probability of 1/2. When the occupational probability of the other sites is 1, the 4D model leads to a Ba:Ti:O ratio of 2(3)^1/2^:6 + 2(3)^1/2^:6 + 5(3)^1/2^, which is in good agreement with the stoichiometry of Ba_0.37_TiO_1.55_ (Cockayne *et al.*, 2016 ▸). The atomic arrangement of the Ba–Ti–O DDQC obtained from the 4D model is presented in Fig. 3 ▸(*b*), in which the atomic arrangement was generated with the following parameters: *x*_1_ = *u*_1_ = 1/4, *x*_2_ = *u*_2_ = 1/8, *y*_3_ = *z*_3_ = 3/8, *y*_4_ = *z*_4_ = 1/4 and *y*_5_ = *y*_6_/2 = *y*_7_ = *u*_5_/2 = *u*_6_ = *u*_7_ = 1/8.

Point density of the QC is calculated from the ODs and the unit-cell volume of the higher-dimensional structure. In the 4D model of the DDQCs, the unit-cell volume is given by det|*Q*_*ij*_|, where *Q*_*ij*_ is the 4 × 4 matrix in equation (2). The point density is then given by ρ = *V* 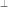 /det|*Q*_*ij*_|, where *V* 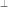 is the sum of the area of the ODs (Yamamoto, 1996 ▸). Because the point density of Ba in the 4D model equals 3^1/2^/*a_d_* ^2^, the number densities for Ba, Ti and O are approximately 2.46 × 10^−2^, 6.72 × 10^−2^ and 10.4 × 10^−2^ Å^−2^, respectively. According to the STM observation, the areal density of the protrusions for the Ba–Ti–O DDQC is 3.2 × 10^−2^ Å^−2^ (Yuhara *et al.*, 2020 ▸). This density is rather close to the point density of the Ba in the 4D model.

Yuhara *et al.* (2020 ▸) recently reported the number densities of Ba, Ti and O for the Ba–Ti–O DQC, which were determined to be (8 ± 3) × 10^−2^, (4 ± 2) × 10^−2^ and (3 ± 1) × 10^−2^ Å^−2^, respectively. A similar result was obtained by the same group in a more recent study where the Ba–Ti–O DDQCs formed from three different precursor Ba–O thin films were investigated (Li *et al.*, 2021 ▸). However, the experimental atomic density was inconsistent with the 4D model for, *inter alia*, the following reasons: first, the reported atomic density of O is significantly lower than the atomic density of O expected from the 4D model, and second, the reported atomic density of Ba is significantly higher than the atomic density of Ba expected from the 4D model. The first is explained by the presence of an oxygen defect results from low oxygen partial pressure during the annealing of the ultra-thin film. The second cannot be explained and remains an open question. To explain the large deviation between the ex­peri­mental and expected atomic densities, the atomic occupation probability must be refined, together with the positions of each OD of the 4D model in the structure refinement using the SXRD intensities. According to the SXRD experiment on the Ba–Ti–O DQC (Schenk *et al.*, 2019*a* ▸), only ten independent reflections were observed, which is insufficient for the structure refinement. Therefore, the structural perfection of the Ba–Ti–O DDQC must be improved in order to increase the number of observed reflections in the SXRD experiment, and this is a current research task in progress.

## 4. Approximants

APs are important crystals for understanding the atomic structure of the QCs (Elser & Henley, 1985 ▸). The atomic structure of an AP is derived from a higher-dimensional structure of a QC by the introduction of an appropriate linear phason strain, and the AP exhibits a local structure similar to the QC [see, for example, Yamamoto (1996 ▸), Quiquandon *et al.* (1999 ▸), and references therein]. The formation of several long-range ordered structures with large unit cells has been found to date in Ba–Ti–O ultra-thin films (Förster *et al.*, 2012 ▸). Two types of APs in the (Ba,Sr)–Ti–O ultra-thin films were reported recently, and these consist of three of the NGT tile elements, *i.e.* square, triangle and rhombus. The first is the sigma-phase approximant, which corresponds to an Archimedean tiling (3^2^.4.3.4) composed of squares and triangles, and it was identified in a Ba–Ti–O ultra-thin film on Pt(111) and Ru(0001) (Roy *et al.*, 2016 ▸; Förster *et al.*, 2016 ▸; Zollner *et al.*, 2020*b* ▸). The second has a complex structure composed of squares, triangles and rhombuses with a large unit cell (*a* = 25.1, *b* = 37.7 Å and γ = 95.1°), and it was identified in an Sr–Ti–O ultra-thin film (Schenk *et al.*, 2017 ▸). Here, we present two of the simplest APs derived from the 4D model by the introduction of linear phason strain.

**Figure 4:**
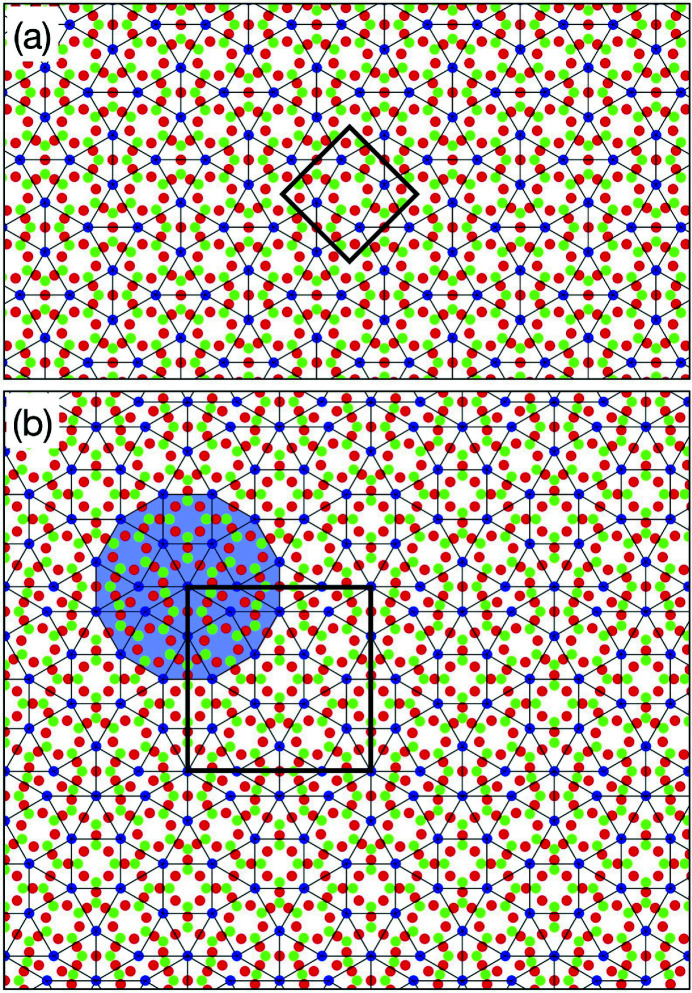
Atomic structure of the Ba–Ti–O APs derived from the 4D model. (*a*) The sigma-phase approximant. (*b*) The hypothetical AP with a unit-cell parameter of 25.6 Å. Circles drawn in lime, red and blue indicate Ti, O and Ba atoms, respectively. The thin black square indicates the unit cell. A dodecagon characteristic to the NGT is highlighted by the translucent blue colour.

Fig. 4 ▸(*a*) shows the atomic arrangement of the Ba–Ti–O sigma-phase approximant (plane group: *p*4) by the introduction of a linear phason strain represented by a 2 × 2 phason matrix *U* with *U*_11_ = *U*_22_ = (3^1/2^ − 1)/(3^1/2^ + 1) and *U*_12_ = *U*_21_ = 0. Here, the arrangement was calculated with the parameters used to generate Fig. 3 ▸, which results in a stoichiometry of Ba_0.33_TiO_1.50_. The structure consists of squares and triangles with a lattice constant equal to *a*_*d*_[1 + 1/3^1/2^] ≃ 13.2 Å. The resulting atomic arrangement corresponds to the Y-rows structure proposed by Cockayne *et al.* (2016 ▸). On the other hand, the observed sigma-phase approximant (plane group: *p*2, *a* = 13.1, *b* = 12.9 Å and γ = 90.5°) (Roy *et al.*, 2016 ▸) is slightly distorted and exhibits a symmetry lower than the derived structure. The distorted structure can be derived by shearing the 4D model along the 2D 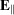 , in addition to the phason strain.

Fig. 4 ▸(*b*) shows the atomic arrangement of a hypothetical AP (plane group: *pm*) by the introduction of a linear phason strain represented by a phason matrix *U*, with *U*_11_ = *U*_22_ = (3^1/2^ − 2)/(3^1/2^ + 2) and *U*_12_ = *U*_21_ = 0. The structure consists of three tiles, *i.e.* triangle, square and rhombus, and the dodecagon characteristic to the NGT is also observed in the structure. The stoichiometry is TiO_1.62_Ba_0.38_ and the lattice constant is 2*a*_*d*_(3^1/2^ + 2)/6^1/2^ (≈ 25.6 Å). The resulting atomic arrangement corresponds to the 25.6 Å approximant in the literature (Cockayne *et al.*, 2016 ▸); however, this structure has not been found experimentally.

We note that the complex AP (*a* = 25.1, *b* = 37.7 Å and γ = 95.1°) in the Sr–Ti–O ultra-thin film (Schenk *et al.*, 2017 ▸) is derived from a linear phason strain represented by a phason matrix *U* with *U*_11_ = *U*_22_ = (3^1/2^ − 2)/(3^1/2^ + 2), *U*_12_ = −2(3)^1/2^/{3[4(3)^1/2^ + 7]} and *U*_21_ = 0. The calculated lattice constant are *a* = 2*a*_*d*_(3^1/2^ + 2)/6^1/2^ ≃ 25.6 Å, *b* = 2*a*_*d*_[9(3)^1/2^ + 16]^1/2^/6^1/2^ ≃ 38.5 Å and α = 95.1°, which are close to the experimental lattice constant.

## 5. Summary

We have derived the 4D model for the Ba–Ti–O DDQC by considering a tile model of the DDQC. 4D coordinates of the asymmetric part of the ODs were also provided in the 4D model. The atomic structure of the Ba–Ti–O DDQC results in a Ba:Ti:O ratio of 2(3)^1/2^:6 + 2(3)^1/2^:6 + 5(3)^1/2^, which corresponds to a stoichiometry of Ba_0.37_TiO_1.55_. In addition, the point density of Ba atoms in the 4D model was shown to be 3^1/2^/*a_d_* ^2^ ≈ 2.46 × 10^−2^ Å^−2^, which is in good agreement with the observed areal density of the protrusions on the STM images.

Lastly, we have shown that the atomic arrangement of the sigma-phase approximant and the hypothetical AP are derived from the 4D model by the introduction of a linear phason strain, and these structures consist of the same tile arrangement as in the Ba–Ti–O DDQC model.

## Figures and Tables

**Table 1:** Atomic positions of the 4D model for the simple NGT in Fig. 2 ▸, with the Wyckoff positions (W. S.), site symmetry and coordinates, and the OD in Fig. 2 ▸ is listed in the fourth column

W. S.	Site symmetry	Coordinates	OD
1*a*	12*mm*	(0,0,0,0)	a
6*a*	*mm*2	(0,1,0,0)/2	b
4*b*	3*m*	(0,2,0,1)/3	c
3*a*	*mm*4	(0,1,1,0)/2	d
6*b*	*mm*2	(1,0,0,1)/2	e

**Table 2:** 4D positional vectors **x** that define the asymmetric part of the ODs in Fig. 2 ▸, with the vectors presented in units of 2*a*_*d*_/6^1/2^

**Vectors that define the asymmetric part of the OD in Fig. 2 ▸(*a*)**
(OD a1)
**x**_1_ = (0, 0, 0, 0) 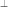
**x**_2_ = (0, 3^1/2^ − 1, 0, 0) 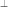 /2
**x**_3_ = (3 − 2(3)^1/2^, 2(3)^1/2^ − 3, 0, 0) 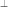 /3
(OD a2)
**x**_1_ = (3^1/2^ − 2, 2 − 3^1/2^, 0, 0) 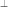
**x**_2_ = (3 − 2(3)^1/2^, 2(3)^1/2^ − 3, 0, 0) 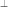 /3
**x**_3_ = (0, 3^1/2^ − 1, 0, 0) 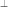 /2
(OD a3)
**x**_1_ = (0, 1, 0, 0) 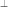
**x**_2_ = (3^1/2^ − 2, 2 − 3^1/2^, 0, 0) 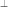
**x**_3_ = (3^1/2^ − 3, 3 − 3^1/2^, 0, 0) 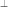 /3
**x**_4_ = (0, 3^1/2^ − 1, 0, 0) 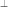 /2
(OD a4)
**x**_1_ = (0, 1, 0, 0) 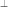
**x**_2_ = (−3^1/2^, 3^1/2^, 0, 0) 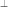 /3
**x**_3_ = (3^1/2^ − 3, 3 − 3^1/2^, 0, 0) 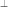 /3

**Vectors that define the asymmetric part of the OD in Fig. 2 ▸(*b*)**
(OD b)
**x**_1_ = (0, 1, 0, 0) 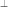 /2
**x**_2_ = (−3^1/2^, 3^1/2^, 0, 0) 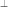 /3
**x**_3_ = (0, 1, 0, 0) 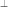
**x**_4_ = (0, 0, 0, −1) 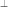
**x**_5_ = (−3^1/2^, 0, 0, −3^1/2^) 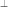 /3
**x**_6_ = (−1, 0, 0, 0) 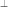

**Vectors that define the asymmetric part of the OD in Fig. 2 ▸(*c*)**
(OD c1)
**x**_1_ = (0, 2, 0, 1) 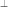 /3
**x**_2_ = (3^1/2^ − 2, 2 − 3^1/2^, 3^1/2^ − 2, 0) 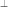
**x**_3_ = (1 − 3^1/2^, 3^1/2^ − 1, 1 − 3^1/2^, 0) 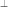 /2
(OD c2)
**x**_1_ = (3^1/2^ − 2, 2 − 3^1/2^, 0, 0) 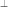
**x**_2_ = (3^1/2^ − 3, 3 − 3^1/2^, 0, 0) 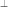 /3
**x**_3_ = (0, 1, 0, 0) 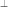
**x**_4_ = (3^1/2^ − 2, 2 − 3^1/2^, 3^1/2^ − 2, 0) 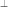
**x**_5_ = (3^1/2^, 6 − 3^1/2^, 3^1/2^, 3 − 3^1/2^) 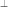 /3
**x**_6_ = (1 − 3^1/2^, 3^1/2^ − 1, 1 − 3^1/2^, 0) 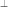 /2
(OD c3)
**x**_1_ = (0, 1, 0, 0) 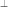
**x**_2_ = (3^1/2^, 6 − 3^1/2^, 3^1/2^, 3 − 3^1/2^) 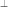 /3
**x**_3_ = (1 − 3^1/2^, 3^1/2^ − 1, 1 − 3^1/2^, 0) 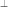 /2

**Vectors that define the asymmetric part of the OD in Fig. 2 ▸(*d*)**
(OD d)
**x**_1_ = (0, 1, 1, 0) 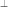 /2
**x**_2_ = (0, 0, 0, −1) 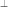
**x**_3_ = (−3^1/2^, 0, 0, −3^1/2^) 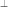 /3

**Vectors that define the asymmetric part of the OD in Fig. 2 ▸(*e*)**
(OD e)
**x**_1_ = (1, 0, 0, 1) 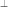 /2
**x**_2_ = (2 − 3^1/2^, 3^1/2^ − 2, 2 − 3^1/2^, 3^1/2^ − 1) 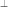
**x**_3_ = (3^1/2^, 0, 0, 3^1/2^) 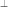 /3

**Table 3:** Occupation domain (OD), atom, coordinates and occupancy (occ.) of the 4D model structure

OD	Atom	Coordinates	Occ.
a1–4	Ba	(0, 0, 0, 0)	1
c1–3	Ti	(0, 2, 0, 1)/3	1
e	Ti	(1, 0, 0, 1)/2 + (*x*_1_, 0, 0, −*u*_1_) 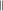	1
e	Ti	(1, 0, 0, 1)/2 − (*x*_1_, 0, 0, −*u*_1_) 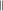	1
e	O	(1, 0, 0, 1)/2 + (*x*_2_, 0, 0, −*u*_2_) 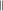	1
e	O	(1, 0, 0, 1)/2 − (*x*_2_, 0, 0, −*u*_2_) 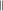	1
d	Ti	(0, 1, 1, 0)/2 + (0, *y*_3_, 0, 0) 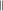	1
d	Ti	(0, 1, 1, 0)/2 − (0, *y*_3_, 0, 0) 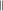	1
d	Ti	(0, 1, 1, 0)/2 + (0, 0, *z*_3_, 0) 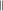	1
d	Ti	(0, 1, 1, 0)/2 − (0, 0, *z*_3_, 0) 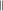	1
d	O	(0, 1, 1, 0)/2 + (0, *y*_4_, *z*_4_, 0) 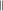	1
d	O	(0, 1, 1, 0)/2 − (0, *y*_4_, *z*_4_, 0) 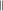	1
d	O	(0, 1, 1, 0)/2 + (0, *y*_4_, −*z*_4_, 0) 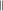	1
d	O	(0, 1, 1, 0)/2 − (0, *y*_4_, −*z*_4_, 0) 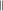	1
c1	O	(0, 2, 0, 1)/3 − (0, *y*_5_, 0, *u*_5_) 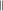	1
c1	O	(0, 2, 0, 1)/3 + (0, *y*_6_, 0, *u*_6_) 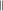	1
c1	O	(0, 2, 0, 1)/3 + (0, −*y*_7_, 0, *u*_7_) 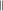	1
c2	O	(0, 2, 0, 1)/3 − (0, 1, 0, 2) 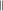 /6	1/2
c2	O	(0, 2, 0, 1)/3 + (0, *y*_6_, 0, *u*_6_) 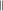	1
c2	O	(0, 2, 0, 1)/3 + (0, −*y*_7_, 0, *u*_7_) 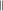	1
c3	O	(0, 2, 0, 1)/3 − (0, 1, 0, 2) 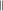 /6	1/2
c3	O	(0, 2, 0, 1)/3 + (0, 2, 0, 1) 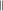 /6	1/2
c3	O	(0, 2, 0, 1)/3 + (0, −*y*_7_, 0, *u*_7_) 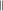	1
